# Summary of the National Advisory Committee on Immunization (NACI) Statement: Updated guidance on human papillomavirus (HPV) vaccines

**DOI:** 10.14745/ccdr.v50i12da01

**Published:** 2024-12-05

**Authors:** Nicole Forbes, Josh Montroy, Marina I Salvadori, Vinita Dubey

**Affiliations:** 1 Public Health Agency of Canada, Centre for Immunization and Respiratory Infectious Diseases, Ottawa, ON; 2 Department of Pediatrics, McGill University, Montréal, QC; 3 Toronto Public Health and University of Toronto Dalla Lana School of Public Health, Toronto, ON

**Keywords:** National Advisory Committee on Immunization, HPV, Canada, 9vHPV, Gardasil-9, cancer, vaccine guidance

## Abstract

**Background:**

Without vaccination, approximately 75% of people in Canada will acquire a human papillomavirus (HPV) infection in their lifetime. HPV vaccine coverage rates continue to fall short of the national goal of 90% coverage for two or more doses by 17 years of age. Recent evidence and World Health Organization (WHO) guidance now support a 1- or 2-dose schedule for younger age groups, which can simplify vaccination efforts and improve coverage rates compared to a multi-dose immunization program.

**Methods:**

The National Advisory Committee on Immunization (NACI) reviewed available evidence on the clinical benefits and risks of a 1-dose HPV vaccine schedule, as well as additional factors, including ethics, equity, feasibility and acceptability. The evidence and programmatic considerations were organized using a process informed by the Grading of Recommendations Assessment, Development and Evaluations (GRADE) framework and all of the information was used to facilitate NACI guidance development.

**Results:**

A 1-dose schedule is highly effective against HPV infection based on available evidence in younger female populations, with current follow-up of up to 11 years following vaccination. Infectious disease modelling shows that a 1-dose strategy in males and females in Canada is expected to have similar health outcomes over the short and long term compared to two doses.

**Conclusion:**

NACI updated recommendations for individuals 9 to 20 years of age to receive one dose of 9vHPV (Gardasil-9, Merck) vaccine. For individuals 21 years of age and older, a 2-dose schedule should be administered. Individuals considered immunocompromised and individuals infected with HIV should receive a 3-dose series. NACI also issued a discretionary recommendation for HPV vaccination for individuals 27 years and older, and updated guidance to allow HPV vaccine during pregnancy.

## Introduction

Human papillomavirus (HPV)-associated diseases pose a substantial public health challenge globally and in Canada. HPV infection is highly prevalent, with an estimated 75% of individuals experiencing at least one infection in their lifetime if unvaccinated ((1)). HPV-associated diseases include cervical, anal, and oropharyngeal cancers, as well as anogenital warts and recurrent respiratory papillomatosis, a rare but serious disease.

In Canada, HPV vaccination is a cornerstone of public health efforts to prevent HPV-related diseases. The Canadian HPV Immunization Program aims to reduce HPV-associated morbidity and mortality by ensuring universal access to vaccines ((2)) and the current national goal is to achieve 90% vaccine coverage of 2 doses or more by 17 years of age ((3)). The goal aligns with HPV vaccination goals set forth in the Canadian Partnership Against Cancer Action Plan for the Elimination of Cervical Cancer in Canada ((4)). However, vaccination rates vary across provinces, with many jurisdictions falling well below the 90% goal ((5))

The National Advisory Committee on Immunization (NACI) last updated its recommendations in 2017, recommending 2- or 3-dose schedules depending on age and immune status. Recent evidence and World Health Organization (WHO) guidance now support a 1- or 2-dose schedule for younger age groups, which is expected to potentially simplify vaccination efforts and improve coverage rates compared to a multi-dose immunization program. According to the WHO 2022 guidance, a single-dose schedule, referred to as an alternative, off-label single-dose schedule, can provide comparable efficacy and durability of protection to a 2-dose regimen for individuals aged 9 to 20 years ((6)).

NACI has since reviewed evidence and provided guidance on the recommended use of HPV vaccines, and updated recommendations on HPV vaccine schedules ((5)). A summary of updated NACI guidance on HPV vaccines follows.

## Methods

For this interim guidance, NACI reviewed key questions as proposed by the NACI HPV Working Group, including those on HPV vaccine schedule by population and on HPV vaccine guidance during pregnancy. Evidence synthesis was performed by the NACI Secretariat and reviewed by the NACI HPV Working Group. Following critical appraisal of individual studies, summary tables with ratings of risk of bias informed by Cochrane RoB 2 and ROBINS-I, as appropriate, were prepared ((7)). The evidence and programmatic considerations were organized by the NACI Secretariat using a process informed by the Grading of Recommendations Assessment, Development and Evaluations (GRADE) framework and all of the information was used to facilitate development of NACI guidance.

NACI uses a published, peer-reviewed framework and evidence-informed methodology to ensure that issues related to ethics, equity, feasibility and acceptability are systematically assessed and integrated into the guidance ((8)). NACI considered feedback provided by the Public Health Ethics Consultative Group, the Canadian Immunization Committee and the Public Health Agency of Canada. Further information on NACI’s evidence-based methods is available in *Evidence-based recommendations for immunization - Methods of the National Advisory Committee on Immunization*.

To inform policy recommendations in Canada, mathematical modelling was used to project the population-level impact and efficiency of switching from 2-dose to 1-dose gender-neutral routine HPV vaccination ((9)). Using the previously validated HPV-ADVISE model, an individual-based transmission-dynamic model of HPV infection and disease, two provinces (Québec and Ontario) were modelled; these two provinces represented higher (≈85%) and lower (≈65%) HPV vaccination coverage in Canada, respectively. Non-inferior and pessimistic scenarios of 1-dose efficacy (vaccine efficacy=98%, 90%) and average duration of protection (duration of vaccine protection=lifelong, 30 years, 25 years) were compared to two doses (vaccine efficacy=98%, duration of vaccine protection=lifelong). The main outcomes were incidence of HPV-16 infections (among females and males), cervical cancer and other HPV-associated cancers and the number needed to vaccinate to prevent one case of cervical cancer.

NACI reviewed available evidence and approved updated guidance on May 27, 2024.

## Results

Efficacy and effectiveness of a 1-dose HPV vaccine schedule compared to no HPV vaccine

Compared to no HPV vaccine, the available evidence from randomized controlled trials demonstrated that a 1-dose HPV vaccine schedule resulted in a large reduction in persistent HPV infections with product-specific vaccine types, through three years following vaccination (high certainty of evidence) ((10)). Evidence from non-randomized trials demonstrated similar effects, with a single dose of HPV vaccine resulting in reductions of persistent, incident and prevalent HPV infections with product-specific vaccine types, compared to no vaccine (moderate certainty of evidence; follow-up ranging from 6 to 11 years) ((11–14)), as well as reductions in anogenital warts (moderate certainty of evidence; follow-up of approximately 2.5 years) ((15)).

Efficacy and effectiveness of a 1-dose HPV vaccine schedule compared to a 2- or 3-dose HPV vaccine schedule

Compared to a 2- or 3-dose schedule, available evidence suggests that a 1-dose schedule may provide similar protection from HPV infection with product-specific vaccine types, through 11 years following vaccination. Compared to two or three doses, there may be little to no difference in the risk of persistent, incident or prevalent HPV infections with product-specific vaccine types (low certainty of evidence, follow-up ranging from 4 to 11 years) ((11–13)), or in the risk of anogenital warts (low certainty of evidence; follow-up of approximately 2.5 years), with a 1-dose HPV vaccine schedule ((15)). Similarly, there may be little to no difference in the risks of cervical abnormalities or cervical intraepithelial neoplasia grade 2+ (CIN2+) between 1-dose and either 2- or 3-dose schedules (low certainty of evidence; follow-up of 10 years), although evidence is currently limited ((12)).

### Immunogenicity

Numerous clinical trials have demonstrated a 1-dose, 2-dose or 3-dose HPV vaccine series generates a robust immunological response to HPV vaccine antigens. While a 2- or 3-dose schedule results in significantly higher antibody titers than a 1-dose schedule, the response generated by a 1-dose, 2-dose or 3-dose HPV vaccine schedule first peaks, then remains relatively stable, out to 16 years. Compared to natural infection, a single dose results in significantly higher antibody titres, out to at least 10 years ((16,17)). Currently, there is no established correlate of protection for HPV, and therefore the clinical relevance of differences in the immune response following different HPV vaccine schedules is unknown ((17–19)).

### Vaccine safety

According to 9vHPV clinical trial data, the most common injection-site reactions following vaccination in those 9 to 26 years of age were pain, swelling, and redness. The most common systemic reactions included headache and fever (37.8°C or greater) for both sexes, as well as nausea for females. Female participants reported higher frequency of adverse events following the third dose of 9vHPV compared to the first two doses for all outcomes, except any pain, which was highest following the second dose in females aged 16 to 26 years ((20)). For males, injection site adverse events were generally similar after the first, second and third doses; however, the frequency of vaccine-related systemic events was highest following the first dose and decreased following subsequent doses ((21)). Additionally, no safety concerns associated with the 9vHPV vaccine have been identified with the Canadian Adverse Events Following Immunization Surveillance System (CAEFISS) from product licensure date to time of NACI guidance deliberations.

Regarding 9vHPV vaccine safety during pregnancy, available data indicate no increased risk of adverse pregnancy outcomes associated with 9vHPV during or around pregnancy, and any adverse outcomes appear to occur at similar rates as observed in the general population ((22)), which is consistent with the safety profile for 2vHPV (bivalent HPV vaccine; Cervarix, Glaxo-Smith-Kline) and 4vHPV (quadrivalent HPV vaccine; Gardasil-4, Merck) vaccines when administered during pregnancy ((23)).

### Disease modelling

Canadian-specific disease modelling estimates that 1-dose HPV vaccination would avert a number of HPV-associated cancers that is similar to two doses in Canada, under various modelling scenarios ((9)). Additionally, all 1-dose strategies were projected to lead to cervical cancer elimination (fewer than four cases per 100,000 woman years) within 15 to 25 years and were projected to be a substantially more efficient use of vaccine doses compared to two doses (number needed to vaccinate for 1-dose vs. no vaccination: 768 to 1,012 individuals; incremental number needed to vaccinate for 2-dose vs. 1-dose vaccination: >10,000 individuals). Switching to a 1-dose program nationally would still produce gains despite varying vaccination coverage among provinces. If 1-dose protection is shown to wane substantially in the next 10 years, modelling showed that switching back to 2-dose routine vaccination of adolescents after 10 years of 1-dose vaccination could mitigate losses in HPV-associated cancer prevention, leading to similar numbers of cancers averted as would be the case in remaining with 2-dose vaccination (catch-up vaccination with a second dose would not be required) ((9)). Additional work is required to better understand the progression rate and dynamics for other HPV-associated cancers and among equity-deprived populations.

### Ethics and equity considerations

Following implementation of a 1-dose HPV immunization program, it will be essential to maintain immunization opportunities to prevent lower vaccination rates among vulnerable communities already facing disparities in HPV-associated diseases. Specifically, First Nations, Métis and Inuit populations in Canada experience higher rates of HPV infection and associated disease, as well as lower cervical cancer screening rates, which can be complicated by stigmatization and discrimination when accessing healthcare ((24)). Of note, recent Canadian data reports that Indigenous women are two to 20 times more likely to be diagnosed with cervical cancer compared to non-Indigenous women and have a mortality rate from cervical cancer four times higher than non-Indigenous women ((25–28)). Immigrant and refugee populations also face increased HPV-related risks and are reported to have lower cervical cancer screening rates ((29)). Addressing socio-demographic disparities in vaccination rates will be integral for equitable HPV immunization policy and may require enhanced access and uptake strategies tailored to equity-denied groups. These may include catch-up programs, expanded vaccine access in primary healthcare delivery and in schools, simplified consent processes and targeted resource allocation to equity-denied groups.

NACI recommendations on HPV vaccines for public health program-level decision-making

The following are recommendations for provinces/territories making decisions for publicly funded immunization programs:

· NACI continues to recommend HPV vaccination for all individuals 9 to 26 years of age. **(*Strong NACI recommendation*)**

· NACI recommends that individuals 9 to 20 years of age should receive one dose of HPV vaccine, and individuals 21 to 26 years of age should receive two doses of HPV vaccine. **(*Strong NACI recommendation*)**

· Nonavalent 9vHPV vaccine should be used, as it provides protection against the greatest number of HPV types and associated diseases. **(*Strong NACI recommendation*)**

### Recommendations for individual-level decision-making

The following recommendation is for healthcare providers advising individual clients:

· Individuals 27 years of age and older may receive the HPV vaccine with shared decision-making and discussion with a healthcare provider. The vaccine should be given as a 2-dose schedule with doses administered at least 24 weeks apart. **(*Discretionary NACI recommendation*)**

### Additional guidance on HPV vaccines

Additional guidance on HPV vaccines includes the following:

· A 2-dose schedule may be considered on an individual basis for individuals 9 to 20 years of age with their healthcare provider. When two doses are offered, doses should be administered at least 24 weeks apart.

· HPV vaccines can be offered in pregnancy; routine questioning about last menstrual period or pregnancy is not required or recommended before offering the HPV vaccine. The rationale for this is as follows:

o HPV infection during pregnancy may lead to adverse outcomes to the pregnant woman or pregnant individual and to the fetus.

o The HPV vaccine is expected to provide a benefit to anyone who is at ongoing risk of HPV infection, including during pregnancy.

o Evidence to date demonstrates no increased risk of adverse pregnancy or fetal outcomes associated with HPV vaccination during pregnancy. There is no known evidence nor biological mechanism to expect an increased risk of adverse pregnancy or fetal outcomes with HPV vaccination during pregnancy.

· NACI reiterates its current guidance on a 3-dose schedule for individuals who are considered immunocompromised, as well as individuals living with HIV, when recommended to receive HPV vaccination. See the *Canadian Immunization Guide* for additional guidance.

· NACI emphasizes the ongoing need for additional public health measures, such as HPV infection and associated cancer screening and surveillance and early access to treatment to prevent HPV-associated diseases for all Canadians. Trends in HPV infection incidence or the incidence of HPV-associated outcomes will be important to monitor in relation to any changes to HPV vaccine immunization programs.

· NACI also encourages dedicated efforts to implement such measures to equity-denied groups, including First Nations, Inuit and Métis people, some of whom face disproportionately high rates of HPV-associated cancers and lower rates of HPV immunization. Continuing to provide HPV vaccination in school-based programs has been shown to reduce health inequities.

Given ongoing efforts to improve HPV vaccination coverage and reduce HPV-associated burden of disease among people in Canada, and considering recent updated guidance from the WHO, NACI used an evidence-informed approach to update guidance on HPV vaccine schedules. NACI guidance on recommended schedules for HPV vaccines is summarized below in **Table 1**. Updated NACI guidance now includes recommendations on the use of the 9vHPV vaccine to provide protection from the greatest number of vaccine-preventable strains. NACI also issued a discretionary recommendation for HPV vaccination for individuals 27 years and older, and updated guidance to allow HPV vaccine during pregnancy.

**Table 1:** NACI recommendations on HPV immunization schedules

Group(s)	NACI guidelines on HPV immunization schedules
9–20 years^a^	1-dose^b^ HPV vaccine schedule with 9vHPV
21–26 years^a^	2-dose HPV vaccine schedule with 9vHPV; doses administered at least 24 weeks apart
27 years and older^a^	2-dose HPV vaccine schedule with 9vHPV; doses administered at least 24 weeks apart
9 years and older^a^ who are immunocompromised or living with HIV	3-dose HPV vaccine schedule^c^ with 9vHPV

Abbreviations: HPV, human papillomavirus; NACI, National Advisory Committee on Immunization

^a^ Recommended schedule is based on age at initiation of vaccination

^b^ A 2-dose schedule may be considered on an individual basis for individuals 9–20 years of age. When two doses are offered, doses should be administered at least 24 weeks apart

^c^ Individuals recommended to receive HPV vaccine who are immunocompromised, including individuals living with HIV, should receive a 3-dose HPV vaccine schedule with a nonavalent HPV vaccine. The minimum interval between the first and second doses of vaccine is four weeks (one month), the minimum interval between the second and third doses of vaccine is 12 weeks (three months) and the minimum interval between the first and last doses is 24 weeks (six months)

Note: Refer to the *Human papillomavirus (HPV) vaccines: Canadian Immunization Guide* chapter in Part 4 for additional guidance on recommended HPV vaccine schedules

## Conclusion

NACI will continue to monitor evidence of 1-dose HPV vaccine schedules, including long-term durability (e.g., 20+ years follow-up time) and clinical outcomes. As evidence becomes available from clinical trials, Canadian data and other countries where similar schedules are adopted, NACI will issue updates to guidance as warranted. To maximize the benefits of a reduced dose schedule, Canadian jurisdictions that adopt 1-dose HPV immunization programs should aim to increase coverage and maintain immunization opportunities among those at risk, especially among vulnerable communities already facing disparities in HPV-associated diseases.
